# Current strategies with implementation of three-dimensional cell culture: the challenge of quantification

**DOI:** 10.1098/rsfs.2022.0019

**Published:** 2022-08-12

**Authors:** Jonathan Temple, Eirini Velliou, Mona Shehata, Raphaël Lévy, Priyanka Gupta

**Affiliations:** ^1^ Bioscience building, University of Liverpool, Liverpool L69 3BX, UK; ^2^ Centre for 3D Models of Health and Disease, University College London, London, UK; ^3^ Hutchison-MRC Research Centre, University of Cambridge, Cambridge CB2 1TN, UK; ^4^ Laboratoire for Vascular Translational Science, Université Sorbonne Paris Nord, Bobigny, France

**Keywords:** spheroid, scaffold, organoid, three-dimensional cell culture, cell culture, hydrogel

## Abstract

From growing cells in spheroids to arranging them on complex engineered scaffolds, three-dimensional cell culture protocols are rapidly expanding and diversifying. While these systems may often improve the physiological relevance of cell culture models, they come with technical challenges, as many of the analytical methods used to characterize traditional two-dimensional (2D) cells must be modified or replaced to be effective. Here we review the advantages and limitations of quantification methods based either on biochemical measurements or microscopy imaging. We focus on the most basic of parameters that one may want to measure, the number of cells. Precise determination of this number is essential for many analytical techniques where measured quantities are only meaningful when normalized to the number of cells (e.g. cytochrome p450 enzyme activity). Thus, accurate measurement of cell number is often a prerequisite to allowing comparisons across different conditions (culturing conditions or drug and treatment screening) or between cells in different spatial states. We note that this issue is often neglected in the literature with little or no information given regarding how normalization was performed, we highlight the pitfalls and complications of quantification and call for more accurate reporting to improve reproducibility.

## 1. Introduction

**Figure 1.**
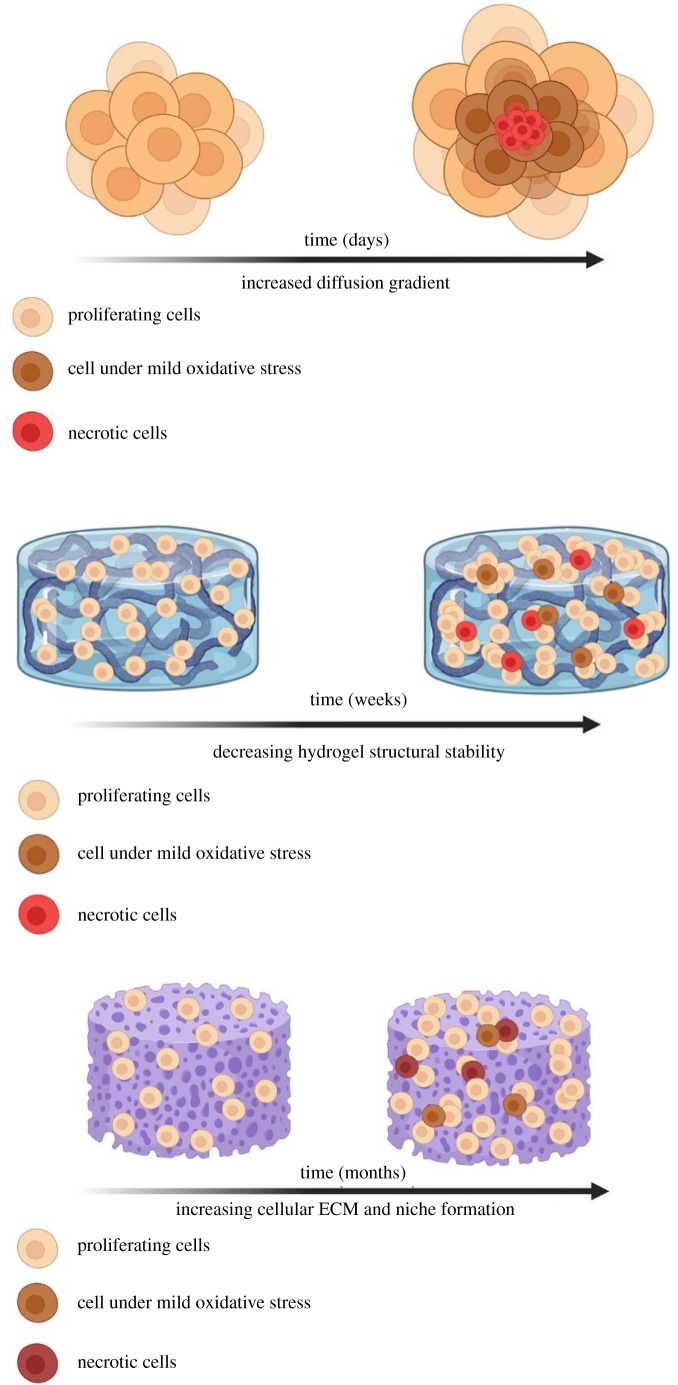
The heterogeneity of cells cultured in different 3D systems: spheroids, hydrogels and scaffolds. Created using BioRender.

The importance of three-dimensional (3D) cell culture techniques to mimic *in vivo* conditions has been known since the 1980s but it has not been until the last decade with the advancement of understanding, biomaterial development and technology that the field has taken off [1,2]. The increase in research has led to a variety of 3D culture systems that have been developed to make cell cultures more representative of physiological conditions including spheroids, sandwich cultures and cells growing on hydrogels or polymer scaffolds, most of which can be made dynamic by incorporation into bioreactors or put under flow. Growing cells is a key activity of most research laboratories across the world, and 2D cell culture is still the benchmark for most fields including pharmaceutical testing [3–6]. This is in part due to the lack of established robust and reproducible protocols and the difficulties in attaining accurate biochemical readings and images in 3D culture systems. The huge variation in both size and structural and biochemical complexity between different 3D systems (figure 1) further complicates the task of comparing their performance in replicating the physiological environment.

## 2. Types of 3D cell cultures

The 3D cell culture technologies can be broadly grouped into scaffold-free and scaffold-based. The former relies on low adherence surfaces to encourage cells in suspension to aggregate through techniques including hanging-drop, low adherence round bottom wells and rotating cultures. These cultures self-organize, produce and organize their own extracellular matrix (ECM) just like *in vivo* tissues and form extensive cell-to-cell contacts making them akin to the avascular *in vivo* environment of organs, including the heart, liver, eye and pancreas [7–10]. The culturing of scaffold-free cultures is a simple and cheap process, even possible in Petri dishes [11,12] while being scaleable, enabling the mass production of spheroids of a uniform and controllable size for high-throughput applications [12–14]. By contrast, scaffold-based cultures, including hydrogels and polymer scaffolds, use a physical network to mimic the ECM of the tissue; providing a substrate for cells to interact with. These may be fabricated from synthetic or natural materials and are customizable in terms of shapes, size and biomolecular cues to best mimic the structure of *in vivo* tissue.

A stepping stone between 2D and 3D, sandwich cultures have been established for three decades [15] whereby a 2D cell layer is placed between thin layers comprising ECM proteins. They provided an early demonstration of the benefits of more complex culture systems, for example, hepatocyte activity and function were much more analogous to hepatocytes *in vivo* [16–19].

Similar in architecture to sandwich cultures, hydrogels are water-swollen networks of cross-linked polymers, typically ECM components that completely suspend the cells in ECM offering them a 3D environment. These gels can be natural or synthetic, with natural hydrogels formed of proteins and ECM components such as collagen, chitosan or Matrigel [20–22]. They are known to be biocompatible, non-immunogenic and have been shown to enhance multiple cellular activities [20,23,24]. Synthetic gels are often composed of materials such as poly(ethylene glycol) [25,26] or polyacrylamide [27,28] which boast simple chemistry and robust manufacturing, while being highly customizable for further applications.

Polymer scaffolds can be made from both natural or synthetic materials and, depending on their composition, replicate different features of tissue ECM including internal organization and mechanical stiffness. Polymeric scaffolds are fabricated through various methods, such as gas foaming, electrospinning, lyophilization and 3D printing. Depending on the polymer and fabrication method selected, a variety of scaffolds with different internal organization, pore size, geometry and overall elasticity can be generated. Typically, natural polymers lead to softer scaffolds and synthetic polymers to scaffolds with higher stiffness, including sponge-like structures. Overall, the versatility of biomaterials and fabrication techniques enables the generation of a wide range of structures with controlled properties that can mimic different tissues and diseases [29–36].

To improve these 3D cultures, both scaffold-free and scaffold cultures can be incorporated into bioreactors. Bioreactors exist in several designs, including spinning flasks, rotating walls, perfusion systems, capillary fibres and chip devices. They all aim to overcome one of the main limiting factors of other 3D culture systems, nutrient and gaseous exchange. The cells in these systems are typically under sheer stress, allowing a high mass transfer rate to be achieved throughout the cultures [37–39]. Bioreactors have been successfully implemented for a variety of cell types, including heart, muscle, liver, embryonic stem cells and mesenchymal stem cells [40–45]; while showing great potential in the toxicity testing of potential therapeutic drugs [46–48].

Further to these different 3D cultures, to accurately study normal/disease phenotypes and heterogeneity, many are turning towards more complex 3D systems. The advances of culturing mini organs outside of the body have made it possible to use 3D culture techniques as an alternative to *in vivo* models. These mini organ cultures, termed ‘organoids', originate from a variety of sources including neonatal tissues, pluripotent/induced pluripotent stem cells, tissue biopsies and adult stem cells. The resulting organoids self-organize and recreate the physiology of organs, as well as accurately represent clinical diseases in remarkable detail [49].

Organoids initially were used to model tissue development and stem cell fate. Genes of interest were marked or removed, and the resulting organoids followed in real time to identify lineage specifications and cell fates [50–53]. Today organoids can be used in many experimental approaches developed for cell lines. The ability to use organoids, especially derived from human tissues, for an array of applications, including disease modelling, regenerative medicine, drug discovery and personalized medicine, has received widespread attention.

With such huge differences in geometry and complexity, analysing and comparing these models is daunting and complicated. Yet, systematic benchmarking of these approaches is a necessity or the field risks stagnation, where new systems are developed for the sake of novelty rather than for the potential benefits they bring to the understanding and modelling of biology. It is also a necessity as these cultures come with experimental hurdles, from reproducible culture protocols to monitoring detailed biochemical information and high-quality cell imaging. Overall, for an accurate establishment of 3D cultures as pre-clinical models for drug or therapy screening, several challenges need to be overcome, particularly with respect to readouts from these systems. Some practical challenges that are generally faced in 3D cultures include: (i) difficulty in extracting the cells from different biomaterial-based 3D constructs due to classic dissociation techniques being inefficient and highly influenced by the structural complexity of the culture; (ii) diffusional limitations and gradient formations of nutrients, gases, reagents, dyes and antibody solutions, which can lead to inaccurate results and problems with imaging; and (iii) the inability to account for the number of cells within the culture as normal cell counting methods rely on obtaining a single-cell suspension and other proxy measures may be influenced by the transition from 2D to 3D. A good overview of the challenges faced is demonstrated within the SWOT (strengths, weaknesses, opportunities and threats) analysis performed by Carragher *et al*. [54]. Although specific to high-content analysis, all points are relevant to the field of 3D cell culture.

## 3. Making the transition from 2D to 3D

Is 3D better than 2D? While this is often a starting argument, in the absence of a standardized criterion to distinguish 2D from 3D, it is useful to step back and consider the aim that our scientific community is trying to achieve: improve culture systems by making them more representative of the physiological environment. With this in mind, ‘2D' is a shorthand for traditional cell culture on Petri dish while ‘3D’ is any alternative culture system that gets us closer to that aim. This allows us to focus, not on the fraught question of whether a proposed system is 2D or 3D, but the extent to which it is representative of physiological or the pathological environment it is trying to recapitulate (table 1). The comparison between models can then study cellular processes and tissue-specific criteria, such as biomechanics, transport of small molecules, cell-to-cell interactions, ECM production and response to pharmacological agents. We suggest that such clarity of purpose in the development of new culture systems would help with side-by-side comparisons and reduce the risk that the field becomes inundated with systems that are ill defined and difficult to compare.

**Table 1.** The variability of key characteristics of cells growing in different environments.

	‘2D' = growing on a flat surface (glass, plastic)	‘3D' = anything more physiological than ‘2D’	organoids	*in vivo*
**diffusion**	unrestricted	limited by culture system	limited—no vascularization	vascularization
**cell-to-cell interactions**	minimal—side-by-side interactions	increased number of interactions	increased; however, similar to ‘3D’	extensive
**cell physiology**	*in vitro*	highly variable depending on the culture type	comparable to *in vivo*	—
**cell shape**	long and flat	more akin to *in vivo*	comparable to *in vivo*	governed by location and function, highly variable
**proteome/genome**	basic expression	improved expression of key proteins and genes	*in vivo* levels	—

3D cultures have shown potential in a variety of fields including the development of new drugs/drug classes, which require stringent testing and benchmarking. The *in vitro* models used to test drugs, therefore, must be biologically relevant and highly robust. 3D cell culture demonstrated potential to make the process more effective and efficient at the pre-clinical level to reduce animal research, prevent wasted clinical trials and high attrition rates. However, this promising area of application has not seen rapid and extensive uptake of 3D into drug discovery and drug safety evaluation pipelines. This is explained in part by the tight regulation in the pharmaceutical industry but also the characterization challenges associated with these systems.

Organoids also have shown great potential in the study of human biology and disease due to their ability to self-organize, allowing them to recapitulate the physiology and architecture of organs in great detail. Organoids are, therefore, a powerful tool enabling in-depth and real-time monitoring of cancer, infectious diseases and inheritable genetic disorders; however, they also face the same difficulties of standardization and quality control as other 3D cultures while having further complications of expense and starting material [52,53,55–57].

## 4. 3D Culture methods: challenges and good practice

‘2D' cell culture is a relatively simple, cheap and robust process with a variety of culture vessels available depending on the intended purpose of the cultures, along with the quantity of cells required. The technique is easy to learn and is not time consuming, with the main consideration being to avoid contamination. They also face no issues regarding the diffusion of nutrients and gases to the cells, as they are grown in a monolayer.

Scaffold-free cultures are also viewed by many as both simple and cost effective but, are not without extra considerations. For example, hanging-drop techniques are simple and cheap in that they can be cultured using Petri dishes. This process, however, is fiddly and can result in the loss of all samples, if knocked or inverted incorrectly, along with being time consuming when setting up, changing media and collecting samples.

Although tricky, hanging-drop techniques allow for defined size control, unlike ultra-low attachment plates and bioreactors, which can result in spheroids of varying sizes. These two techniques, however, allow for easier long-term cultures and at greater numbers, without as high a risk of losing all of the spheroids. It is therefore worth bearing in mind the advantages of the different culture methods depending on the spheroids' intended purpose.

Aside from these issues, scaffold-free cultures also face difficulties when trying to produce co-cultures that accurately mimic the natural architecture of *in vivo* tissue. This is due to issues in controlling the final location of the different cell types, unlike scaffold-based cultures where the different cell types can be seeded periodically. It is difficult to add cells to spheroids in a layer-based system, as they are likely to only attach to the top side of the spheroid, as the bottom is inaccessible and agitation can be tricky, particularly in hanging-drop cultures.

Using any of these scaffold-free methods, the user also faces issues with collection and handling due to their small size and poor mechanical stability, particularly when compared with scaffold-based systems that are often easier to visualize by eye and can potentially be handled physically by tweezers, etc.

Unlike other scaffold-free 3D cell cultures, organoids are mainly cultured in Matrigel^®^ and are subjected to a variety of different growth factors for the differentiation of stem cells or tissue fragments into the desired organ. This approach, in its modern form, was first described by Sato *et al*. [58] and is the primary protocol used in the field. These protocols for organoid establishment and quality control, however, are not standardized across different laboratories, which can lead to variability and difficulties with reproducibility; meaning that it is vital to confirm, using either microscopy or biochemical analysis, that the organoids are in fact what was intended. Organoids are also relatively expensive when compared with culture methods for traditional cell lines and other model organisms like fly or worms, while also facing potential difficulties when obtaining starting material.

Culture of cells on scaffolds is a more complicated process, as cells need to penetrate the scaffold while ensuring a homogeneous cell distribution within the whole matrix. In hydrogel scaffolds such penetration is less of a problem: they involve creating cell/gel suspensions, which once cross-linked hold the cells in a 3D environment in a more homogeneous manner. Other polymeric scaffolds including porous foams, fibrous or tubular scaffolds rely on seeding through addition of a cell suspension with the anticipation that the cells will diffuse and eventually migrate into the scaffold, often called the ‘drop-on' method [59–63]. This approach, however, is often subject to lower cell attachment, penetration and poor and/or less homogeneous scaffold cellularization, with most cells landing and remaining on the top of the scaffold [5,32]. This is problematic as the cell layer prevents the diffusion of nutrients and reagents to the cells that reside inside the scaffold culture. Furthermore, cell growth takes place locally in ‘pockets' of the scaffold and not in a consistent manner. It is therefore important to investigate and characterize the cell interactions and distribution within the scaffold material.

**Figure 2.**
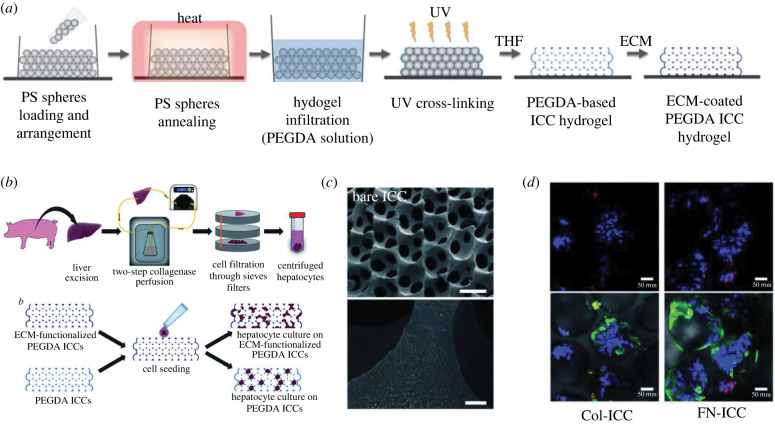
Poly(ethylene glycol) diacrylate (PEGDA) inverted colloidal crystal (ICC) scaffolds prepared by Wu *et al*. [65]. Their figures explain the fabrication of the ICC scaffolds (*a*) as well as how cell seeding was achieved (*b*). SEM imaging of the scaffold shows that the structure indeed looks like the schematic in figure (*c*) and immuno-fluorecence imaging is used to visualize how cells are binding and growing within the structure (*d*).

One way of improving the cell suspension penetration is seeding in dynamic flow and/or with rotation. However, in general the investigation of the cell seeding and cell diffusion/migration into scaffolds is an area which lacks standardization. Many published articles include schematic diagrams, which illustrate the process of scaffold fabrication, but few include the specifics of the culturing process. The latter is particularly important, especially as the cell seeding methods/protocols can be scaffold specific and there is no unique approach to ensure ‘optimal' cell seeding. Consequently, the lack of cell seeding and culturing protocols in publications can make it difficult for readers to understand and appreciate the experimental approach, particularly in cases where little microscopic analysis is performed [64]. Wu *et al.* [65] is an example of good practice with inclusion of informative schematics for both scaffold fabrication and cell culture. This approach clarifies the design of the culture system as well as how cells respond and organize themselves within the material (figure 2) [65]. Such detailed practice is beneficial to other researchers attempting to reproduce or build on the research, as one of the commonly encountered difficulties is seeding or cell distribution after attempting to follow culture methods which lack detailed experimental methodologies and characterization.

Unlike 2D cultures, where plates and flasks are made from standardized tissue culture plasticware, scaffold cultures also face increased variability when using non-commercial, and even to some extent commercial, materials due to non-standardized processes. This can lead to challenges for repeatability and especially reproducibility across different laboratories. Even hydrogels, such as Matrigel, can face issues with batch-to-batch variation potentially affecting results and differences between cultures [66].

Detailed information on the culture method is important, as multiple factors will influence the model, for example, cell seeding concentration, culture length and the technique used for cell seeding. Work by Raghavan *et al*. [67] highlighted that even different spheroid formation techniques affect the end culture. They compared spheroids cultured using three methods: hanging-drop, liquid overlay on ultra-low attachment plates and liquid overlay on ultra-low attachment plates with rotation mixing. Results demonstrated that the spheroids differ in terms of cellular organization and morphology, internal diffusion of nutrients and drugs, ECM deposition and chemosensitivity. It is, therefore, likely that smaller variations in protocols would also results in such differences.

With the increased complexity of 3D culture systems and experimental procedures comes additional technical challenges and experimental optimization. Technical difficulty with 3D cultures, particularly how they are manipulated and handled, is often omitted and yet such crucial information is integral to experimental and data reproducibility. Inclusion of detailed step-by-step protocols is, therefore, something that should be standard practice throughout the field.

## 5. Biochemical analysis: the complications of 3D

As Petri dish-based 2D cell culture has been the gold standard for the past six decades, all current characterization and analysis protocols are tailored to analysing cells in this format [68]. Some biochemical assays have been adapted to 3D, but their application is hindered by the increased complexity in both morphology and functionality of the cultures as highlighted by Fang & Eglen [69]. Difficulties arise due to the hindered diffusion and the entrapment of gases, nutrients, waste and reagents within the systems, along with difficulties when quantifying and normalizing between samples [38,70–74]. For example, spheroid cultures are widely used due to their simplicity and cheap production cost, but they offer little structural support making them difficult to manipulate and handle, while having low porosity for the diffusion of nutrients, gases and assay reagents to the cells. Oxygen can only diffuse through 100–150 µm of tissue, therefore any spheroid above 300 µm is likely to have a hypoxic core [38,72]. As nutrients and assay reagents are far larger than oxygen their diffusion in tissue will be significantly hindered. This will undoubtedly hamper larger cultures such as spheroids over a particular size or scaffolds with low porosity; both in terms of getting nutrients in and waste products out but also for assays which rely on a substrate reaching and being taken up by the cells [3,4]. Organoids, even with their smaller size relative to spheroids, will also experience these issues of diffusion as they are typically grown in a complete matrix like Matrigel, which reduces their permeability.

Even for scaffolds with macro-porosity, after a certain time period, cells form dense and/or large clusters, which can block the pores and lead to uneven distribution and/or diffusional limitations when attempting *in situ* characterization assays. For example, for macroporous polyurethane (PU) foam-type scaffolds which support growth of pancreatic cancer cells, Totti *et al*. [75] has shown little to no differences between different conditions, such as ECM coatings of the scaffolds, when assessing the culture with MTS. By contrast, sectioning, immunostaining and imaging revealed clearer differences between conditions. Similarly, Gupta *et al*. [74] were able to identify differences in viability and/or apoptosis in polymer scaffolds following drug and irradiation screening with advanced microscopy and imaging, in comparison with MTT which was unable to capture differences for different culture conditions. It is therefore important that before beginning analysis of any 3D cultures researchers consider which analytical approach is most appropriate for what they want to study and also how best to normalize across different cultures and conditions. Furthermore, it is important to consider that classical, gold-standard approaches followed in 2D cultures, cannot necessarily be implemented in 3D. For example, when conducting irradiation screening, the gold standard in 2D is the conduction of clonogenic assays for the development of survival curves post-treatment. Hamdi *et al*. [76] highlight the impossibility of extracting cells from spheroids for clonogenic assays and alternatively suggest *in situ* approaches for post-treatment characterization. Such readouts are new and/or differ from standard 2D practice. The field, therefore, needs to consider the most appropriate assay, as what has been validated and accepted for 2D is not always applicable for 3D, depending on the culture type.

To be meaningful, all statistical analysis and cross-comparison biochemical measurements need to be normalized, for example, luminescence data expressed as ‘arbitrary unit value per cell'. However, unlike 2D cultures, counting the exact number of cells in a 3D system is a challenging task. Very few researchers in the field count cells in the traditional manners using microscopy, due to an inability to visualize all the cells within the culture, or using an automated cell counter, as cells can be entrapped within the culture or cannot be collected into a single-cell suspension. The issue is varied across 3D cultures as the more complex the internal organization and chemistry, the more challenging it can be for cell extraction protocols to be successful.

To account for these technical challenges, many researchers report the use of a proxy reading, such as total protein/DNA, to give an estimation of the number of cells within the 3D culture system. These readings theoretically give an accurate estimation of the number of cells; however, they are hindered by reduced reagent diffusion along with some readings, such as luminescence, being affected by the thickness or opaqueness of the culture. Although it is the 3D nature of these cultures that give them enhanced functionality and performance it is also what is causing many of the complications of the associated measurements.

Several proxy measurements are implemented, the simplest being the use of a so-called ‘housekeeping' marker as an indicator of the number of cells. The expression of a protein or gene of interest is reported in relation to a house-keeping gene or protein, such as GAPDH or β-actin. In theory, these markers are constitutively expressed and are required for the maintenance of basic cellular function expression so their levels remain unchanged between cultures and conditions [65,77,78]. Often published as standard practice, this method does not face the same problems as other proxy measurements, such as total DNA/protein concentration, because it is an internal control (the detected levels of both the housekeeping protein/gene and the protein/gene of interest and are affected equally by diffusion and entrapment within samples). However, the problem with this approach is that the expression of both the DNA levels and the protein levels of these housekeeping genes can change, even in 2D, depending on a number of factors including experimental treatment, tissue origin, donor variation, hypoxia and numerous chemical factors, including insulin [77,79–82].

If factors such as hypoxia affect the levels of housekeeping genes/proteins in 2D, then manipulating cells into 3D and other effects associated with some 3D cultures, including being under flow and experiencing shear stress, are likely to also affect their levels. In these cases, using such markers could equally lead to inaccurate measurements between conditions/cultures, particularly when comparing cells cultured in 2D versus 3D, or even between the same 3D cultures if the geometries of the cells are different. This point is highlighted well in the case of β-actin which is a commonly used housekeeping gene. When cells are cultured in 3D, compared with 2D, the expression of components that make up the cell cytoskeleton are altered, seemingly dependent on the tension exerted upon the cells [83]. Work by Pruksakorn *et al.* [84] found that when HepG2 cells are cultured in a scaffold-based 3D culture the expression of cytoskeleton proteins, including β-actin increased when compared with the same cells in 2D. They demonstrate that in these cells the culture geometry has a direct, positive effect on the levels of the housekeeping gene. Conversely, Zhou *et al.* [85] demonstrate that when mesenchymal stem cells are cultured in a 3D spheroid the levels of β-actin decreased dramatically leading to a long-lasting effect on the actin cytoskeleton. They note that it is only the expression of the cytoplasmic β-actin that is reduced, not that of nuclear β-actin expression, and conclude that it is the re-arrangement of the actin cytoskeleton that is largely responsible for the impact of 3D culture on cell size and morphology. A slight decrease in the levels of β-actin was also reported by Kim *et al.* [86] when they cultured the colorectal cancer cell line, SW48, using a 3D soft agar matrix versus 2D. Interestingly, they also reported a dramatic increase in the levels of GAPDH, another commonly used housekeeping marker, compared with the same cells in 2D. This is disconcerting as, although Zhou *et al.* [85] found a large decrease in the level of β-actin, they show that the levels of GAPDH were unchanged between 2D and 3D. The fluctuation in the levels of these housekeeping markers between cells cultured in 2D and 3D is, therefore, highly variable and appears dependent on both cell and culture type. These examples highlight that the use of these housekeeping genes, which is standard practice in 2D, is not necessarily translatable to cells cultured in 3D and should be considered carefully if used.

This method, however, may be suitable when comparing similar 3D cultures to one another, for example, when comparing alike spheroids cultured under different conditions. Alike scaffold cultures, however, may face different levels of these genes due to their higher levels of heterogeneity across samples. It may be possible to prove that levels of certain housekeeping proteins/genes are unaffected between some 3D cultures and 2D or that new housekeeping markers could be identified.

To overcome the challenge of differences in gene expression, the quantification of total protein can be implemented as an alternative approach. Work by Eaton *et al*. [77] demonstrates that using total protein is a more reliable control for quantitative fluorescent Western blotting. The use of total protein or total DNA as a representation of the number of cells in the culture assumes that any change in the expression across different conditions or cultures is negligible at this level. This methodology has been implemented for a long time for different assays from normalizing urea and albumin production [15,64,87–90] to normalizing cytochrome p450 activity [91,92]. Although well characterized and commonly used, this approach is not without its pitfalls; making assumptions that all the cells within the sample have been completely lysed and the internal components released from the culture for measurement. This is problematic due to the likely variation in the levels of diffusion of both lysing agents into 3D cultures as well as the subsequent protein/DNA released, both in spheroid and scaffold cultures. One approach is to firstly homogenize the cultures prior to lysis; however, although possible with spheroids it is not always applicable to scaffold-based cultures, particularly those large in size. Scaffold-based cultures also face complications of protein/DNA retention within scaffold models, which could occur as the result of binding of protein/DNA to the scaffold material or entrapment [70,71]. Like in the case of the housekeeping markers, it is well known that the expression of many proteins/genes differs significantly when comparing cells in 2D and 3D [93–95]. It is, therefore, not unreasonable to suspect that there may also be measurable differences in total DNA/protein expression between samples with the exact same number of cells caused by their culture method. This would mean that using this total protein/DNA as a proxy for cell number for normalization could be inaccurate when comparing 2D and 3D, although, like the housekeeping markers, should be adequate when comparing similar culture methods.

Attempts have been made to increase accuracy and reproducibility of using total protein/DNA. Yan *et al.* [96] demonstrate the use of a standard curve where total DNA was plotted against cell number. The curve was generated by measuring the DNA concentrations from lysates for which the number of cells is known. This idea was first outlined by Feng *et al.* [97] although they do not use it for normalization. While this approach provides readouts as per number of cells, it faces the same issues of diffusion and DNA-scaffold binding. It is possible this approach is less accurate, assuming cells grown in 2D were used to make their standard curves. Additionally, standard curves have also been used for the prediction of cell number through the use of viability [98,99] and proliferation [100], but both would face the same issue of diffusion and binding as Yan *et al.* [96]. Viability and proliferation, however, are not often used to estimate cell number, presumably as they can be directly affected by culture geometry as well as being readouts for other assays such as drug toxicity screening [88,101].

The traditional way in which to normalize across different models and conditions is to count the exact number of cells, without the use of a proxy measurement, ensuring that any measurable difference is down to the functionality of the cells. For 3D cultures, this is more difficult because cell counters and microscopy techniques do not translate well into 3D. Both require all the cells within the culture to be trypsinized and resuspended into a single-cell suspension, something that is easily achieved in 2D due all the cells being on a single, planar surface and accessible by the dissociation solution. However, in 3D it is difficult to ensure all cells are removed from the culture and are in a single-cell suspension, due to extensive cell-to-cell or cell–matrix interactions leading to clumping and entrapment. Despite this, these cell counting methods are still implemented by some researchers in the field [102,103].

It is concerning that, when cell numbers are reported, it is sometimes unclear how it was measured. Using the seeding number of cells [64,65,97] would surely be a significant cause of error as it would assume not only that all cells are taken up into the 3D system but also that there is no change in cell number during the culture time or variation between conditions. Some papers even normalize their albumin and urea readings to cell number without giving any information on how this cell number was calculated [78,104–106]. Often researchers will normalize per well or to a control well, which is effectively normalizing to seeding concentration and has the same pitfalls [107–112]. Four of these studies use 3D spheroid cultures, which in theory should remain consistent in size and cell number if the seeding concentration is the same. However, although more reproducible than other culture types they are not without variation, as can be seen in Ogihara *et al.*'s [107] fig. 2, possibly due to variation in seeding number or variation in growth making normalizing this method less accurate [67,113,114].

Similarly, to other 3D cultures, organoids face the same issues of normalization as mentioned above. They do, however, face further complications as they are heterogeneous in both size and cell population, which leads to complications when trying to measure quantifiable readouts, particularly if normalizing by counting the number of organoids, which is common in the field [115–117].

## 6. Imaging: a powerful tool

**Figure 3.**
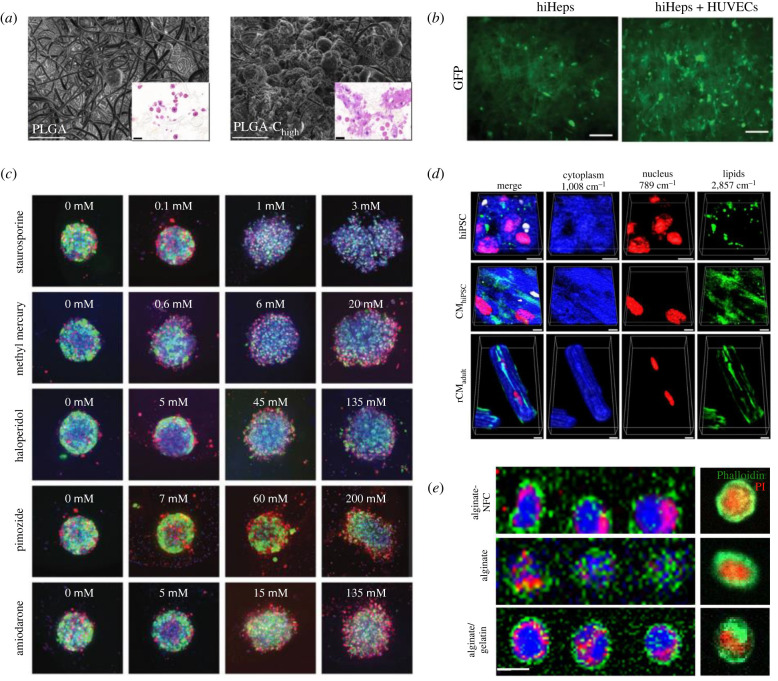
The varying degrees of imaging in the field. (*a*) Electron microscope images demonstrating in high detail how cells integrate with the electrospun fibres [61]. (*b*) Fluorescent imaging shown purely to demonstrate that the cells populate the scaffold and to track growth [106]. (*c*) Quantitative fluorescent imaging of toxicity in spheroids for different drugs [120]. (*d*) Volumetric Raman imaging of cells growing in a hydrogel where the researchers were able to quantify the level of different cell components [121]. (*e*) Raman imaging of spheroids to visualize the distribution of different cell components [122].

Microscopic imaging of 3D systems is a powerful tool that can give a detailed insight into what processes are taking place and to what extent within these systems. Imaging allows a more detailed understanding of the morphological and functional adaptations that the cells undergo when they are cultured in 3D. It allows the visualization of cell distribution throughout the culture and how cells are binding and growing within scaffold materials [35,96], validating whether cells are truly growing in 3D, identification of late stages of differentiation, visualization and semi-quantification of functional markers [118,119] and even toxicity testing [120] (figure 3). Furthermore, obtaining a spatial distribution of the cells in 3D constructs enables the correlation of cell behaviour (proliferation, clustering, secretion of markers, oxidative stress/hypoxia or nutrient stress) to specific structural or biochemical properties of the 3D system. It also allows for mapping/screening of heterogeneity, the latter being critical not only for the validation, understanding and control of 3D cultures, but also for the accurate recapitulation of 3D tissues *in vitro*. Heterogeneity, naturally occurs in healthy and diseased tissues *in vivo* (and it certainly does not occur in traditional 2D cultures), therefore, capturing it and understanding it in 3D is of vital importance.

Imaging cells in 3D can be achieved through physical or optical sectioning, and its importance was noted as early as 1914 [123]. The former involves the mechanical sectioning of the sample to allow imaging deep within the model at high resolution. Although high in resolution and without the hinderance of dye penetration, physical sectioning techniques have limitations; they do not allow real-time imaging due to the sample requiring embedding, and mechanical sectioning can result in the loss of structure [124–126]. Protocols can also be long, time-consuming and arduous, and require sophisticated data reconstruction software.

Optical sectioning offers the potential for quick and non-destructive, three-dimensional imaging of subcellular structures within 3D models. Optical sectioning has been made easier with the development of new technologies like spinning disc and multi-photon microscopy along with light-sheet technologies, allowing greater imaging speed and depth as well as allowing single-plane illumination. These techniques permit imaging of samples in real time without the risk of damage or distortion from embedding and sectioning. Optical sectioning, however, faces other complications that arise mainly due to the penetration of both light and reagents. Huang *et al*. [127] highlight well the different imaging techniques available for imaging 3D cultures and discuss both the penetration depth and resolution of each.

Light penetration will greatly depend on the method of illumination along with opacity and the level of light scattering within the culture; factors that vary across tissues and models [128,129]. Laser-scanning confocal microscopy (LSCM), for example, can penetrate to a depth of approximately 150 µm through brain tissue; however, two-photo microscopy can penetrate more than 500 µm [128,130]. Various techniques exist including classic LSCM, multi-photon and light-sheet illumination, each with different levels of penetration, scattering, bleaching, photo-toxicity and background illumination due to out-of-plane light. The wavelength of the light used will also have an effect, with red light penetrating further than others [129,131]. Light penetration within 3D samples can be improved using clearing, which aims to increase the transparency of the sample and to match the refractive index of the molecules within the tissue to one another. Costa *et al*. [130] give a detailed overview of the different clearing methods and their advantages and limitations. Clearing, however, is only applicable to fixed samples and will only work on biological tissue so will have little effect on cultures using electrospun scaffolds or other such solid matrices.

The main problem with imaging 3D cultures, however, is attaining quantitative data and usually requires the ability to control across samples using cell number. As stated previously oxygen diffusion through tissue is around 150 µm, the diffusion of fluorescent markers and antibodies will be less due to their increased size. The diffusion of these markers is a limiting factor, often more so than light penetration, and will again depend on the 3D model being investigated. The issue here is that without the ability to visualize the whole-cell population it is impossible to attain accurate and reliable data from the whole culture. One solution to this is to just measure from an imageable section; however, due to the natural heterogeneity of most 3D cultures this could lead to unreliable results. Another solution is the use of reporter cell lines that would, therefore, remove the issue of reagent diffusion particularly if using an imaging technique with good light penetration, like multi-photon or light-sheet, and if using cleared samples. Research is also being undertaken in label-free imaging; however, it is still in its infancy for use in 3D applications [121,132–134]. The issue with these techniques, is that without labels to highlight a specific organelle or molecule it is hard to distinguish or study the object of interest. Raman imaging offers a solution here but is difficult to use on 3D cultures due to issues with background signals and poor *z*-axis resolution caused by overlapping signals. Work by Sirenko *et al.* [120] used nuclei staining with Hoechst 33342 as a measure of cell number to normalize compound toxicity, this is one of the few examples in the literature of fluorescent imaging being used quantitatively rather than qualitatively. This approach is informative, as the assay can be multiplexed with other stains used simultaneously to study the cellular pathway the compound effects. The major problem with counting cell number this way, as highlighted by Sirenko *et al.* [120], is that it is very difficult to quantify all of the cells in the 3D system due to problems with light and dye diffusion. They noted how there is a large difference between cells counted and the number of cells seeded, making it difficult to quantify accurately; however, it was a step in the right direction. Interestingly, there has not been much advancement with quantitative fluorescent imaging, probably still due to penetration issues in 3D cultures. Therefore, techniques like Raman imaging are exciting as they could bypass these limitations.

Imaging is informative, yet little to no quantitative measurements are performed using image analysis in 3D cultures, with most imaging work performed only to check cell distribution and binding. This is likely to be the result of microscope and software availability, feasibility of imaging, the paper's intended purpose as well as the factors mentioned above. Often proof that the cells are growing or binding within the culture is all that is required for publication. The quality of imaging is also variable, with some studies imaging using basic confocal or even wide-field imaging. The level of investigation again depends on the need and something that is probably down to cost and availability. Unfortunately, lots of information is being lost when complete analysis of the models is not being undertaken and the inclusion of detailed microscope work, particularly if in 3D, is invaluable in understanding what is going on between different systems, especially when studying co-cultures or demonstrating a new culture system.

## 7. Conclusion

We believe that, if it was possible to establish a standard reporting method, we could face the issues discussed above as a community. Considering the complexity and variability of 3D systems, advanced image analysis is one way to obtain some sort of normalization. One way forward would be to use similar imaging approaches, especially in terms of post-image analysis (unified, advanced computational software that could maximize the quantitative information that can be obtained from an image) and to standardize the approach to conduction of microscopy imaging, for example, how many sections or *z*-stacks are ‘representative' enough. This level of standardization along with the use of biochemical analysis methods that are designed for 3D cultures or have been robustly proven to work for 3D cultures is greatly needed, as traditional 2D cell quantification assays should not just be accepted to work in the 3D field for the reasons highlighted earlier. There are now more companies designing quantification assays for 3D cultures. For example, Promega's line of 3D assays, such as CellTiter-Glo 3D, which are a step in the right direction.

The field of 3D culture shows potential across a wide variety of disciplines; however, we believe that to progress, care and consideration must be implemented. What works for one culture geometry is not necessarily applicable to all, and therefore transparency and detailed methods will improve reproducibility and reduced variability. Further to this, detailed methodologies not only help people within the field already but also aid in the uptake of 3D cell culture into new areas of research. It is important to move away from ‘traditional' and ‘conservative' approaches that are used in 2D culturing, to enable a better understanding and comparison of 3D cultures.

## Data Availability

This article has no additional data.
