# Correction to: ‘Current strategies with implementation of 3D cell culture: The challenge of quantification’ (2022) by Temple *et al.*

**DOI:** 10.1098/rsfs.2022.0066

**Published:** 2023-02-10

**Authors:** J. Temple, E. Velliou, M. Shehata, R. Lévy, P. Gupta

*Interface Focus* **12**, 20220019. (Published online 12 August 2022) (https://doi.org/10.1098/rsfs.2022.0019)

Due to a misunderstanding on part of the primary author, regarding the creation of figure 1, Priyanka Gupta is to be added to the author list. Priyanka created the figure along with Eirini Velliou and, therefore, should be included.

This has now been corrected.

